# Slow chromatin dynamics enhances promoter accessibility to transcriptional condensates

**DOI:** 10.1093/nar/gkab275

**Published:** 2021-04-22

**Authors:** Tetsuya Yamamoto, Takahiro Sakaue, Helmut Schiessel

**Affiliations:** Institute for Chemical Reaction Design and Discovery, Hokkaido University, Kita 21, Nishi 10, Kita-ku, Sapporo 001-0021, Japan; PRESTO, Japan Science and Technology Agency (JST), 4-1-8, Honcho, Kawaguchi, Saitama 332-0012, Japan; Department of Physical Sciences, Aoyama Gakuin University, 5-10-1, Fuchinobe,Chuo-ku, Sagamihara, Kanagawa 252-5258, Japan; Cluster of Excellence Physics of Life, TU Dresden, Dresden 01062, Germany

## Abstract

Enhancers are DNA sequences at a long genomic distance from target genes. Recent experiments suggest that enhancers are anchored to the surfaces of condensates of transcription machinery and that the loop extrusion process enhances the transcription level of their target genes. Here, we theoretically study the polymer dynamics driven by the loop extrusion of the linker DNA between an enhancer and the promoter of its target gene to calculate the contact probability of the promoter to the transcription machinery in the condensate. Our theory predicts that when the loop extrusion process is active, the contact probability increases with increasing linker DNA length. This finding reflects the fact that the relaxation time, with which the promoter stays in proximity to the surface of the transcriptional condensate, increases as the length of the linker DNA increases. This contrasts the equilibrium case for which the contact probability between the promoter and the transcription machineries is smaller for longer linker DNA lengths.

## INTRODUCTION

Enhancers are short regulatory DNA sequences that activate the transcription of target genes. Enhancers are located a long genomic distance (in the order of 10k–1 Mbps) away from target gene promoters and the linker genome thus has to form loops to drive the interactions between enhancers and gene promoters. Mediator complexes that bind to enhancers recruit RNA polymerase II (Pol II) to promoters (1) and promote the assembly of preinitiation complexes (2,3).

Hi-C experiments have shown that genomes of eukaryotic cells are composed of topologically associated domains (TADs) (4–6), which are indeed chromatin loops of the order of 10k–1Mbps (7). The influence of chromatin loops on the promoter–enhancer interactions was studied by molecular dynamics simulations that assume chromatin as a (semi)flexible polymer with static loops at equilibrium (8,9). In contrast to these assumptions, recent theories predict that chromatin loops are produced by the loop extrusion process, with which cohesin acts as a molecular motor that uni-directionally transports chromatin to increase the size of loops until it collides with boundary elements, such as CTCF (10,11). Mediator complexes may also act as boundary elements (11,12). The loop extrusion theory captures the features of the contact frequency map, which is determined by Hi-C experiments (10,11). In earlier single molecule experiments, the motor activity of yeast and human cohesin was not detected (13–15) and alternative mechanisms of loop extrusion process were proposed (16–18). However, the motor activity and the loop extrusion process were directly observed from human and *Xenopus* cohesin in recent single molecule experiments (19,20). It is of interest to theoretically predict the roles played by the dynamics of chromatin looping due to the loop extrusion process in the promoter-enhancer interactions and the regulation of gene expression.

Rao and coworkers used auxin-inducible degron technique, which degrades cohesin in response to a dose of auxin, to eliminate chromatin loops (21). They showed that eliminating chromatin loops did not change the transcription level of most genes (at a time point 6 h after cohesin degradation), but significantly decreases the transcription level of the target genes of superenhancers, which are genomic regions with high density of enhancers (22). Mediator complexes, transcription factors and Pol II form condensates (which are called transcriptional condensates) due to the phase separation driven by the multivalent interactions between the intrinsically disordered domains of these proteins (23–28). Recent microscopic experiments showed that superenhancers colocalize with transcriptional condensates (23,24). Since the linker DNA between the promoters and enhancers is excluded from the transcriptional condensates (26–28), this implies that the superenhancers are localized at their surfaces (23,24). These experiments suggest that the loop extrusion of chromatin at the surfaces of transcriptional condensates plays a key role in enhancing the transcription level of the target genes of superenhancers.

We have therefore theoretically analyzed the dynamics of chromatin, which is extruded by cohesin, at the surface of a transcriptional condensate (29,30). First, by using a bead-spring model we predicted that the mean square end-to-end distance of a chromatin region decreases with a constant rate in the bulk solution, whereas it does not decrease until the tension generated by cohesin, extruding the chain from the grafted end, reaches the free end (29). Second, we used Onsager’s variational approach to predict that the loop extrusion process increases the local concentration of chromatin at the surface of a transcriptional condensate and the lateral pressure generated by the excluded volume interactions between chromatin units decreases the surface tension of the transcriptional condensate (30). Here we take into account the chromatin dynamics driven by the loop extrusion process in an extension of the Langmuir’s theory of surface adsorption to predict the accessibility of gene promoters to the transcription machineries in a transcriptional condensate.

Large transcriptional condensates are stable for the experimental time scale (with which mouse embryonic stem cells are differentiated), whereas the lifetime of small transcriptional condensates is in the order of 10 s (25). For simplicity, we limit our discussion to stable transcripition condensates. Our theory predicts that for cases in which the loop extrusion process is effective, the contact probability increases with increasing the length of linker chromatin. This contrasts with the case in which loop extrusion is inhibited, where the contact probability decreases with increasing the length of linker chromatin between the gene promoter and the enhancer. The increased contact probability in the presence of loop extrusion reflects the fact that the relaxation time, with which the gene promoters stay in proximity to the surface of the transcriptional condensate, increases with increasing the length of the linker chromatin (31,32). The longer relaxation time enhances the probability that the gene promoter rebinds to the surface of the transcriptional condensate. The contact probability of gene promoters to transcriptional machineries is proportional to the length of transcription bursts and may be experimentally accessible.

## MATERIALS AND METHODS

### Chromatin section anchored at surface of transcriptional condensate

**Figure 1.**
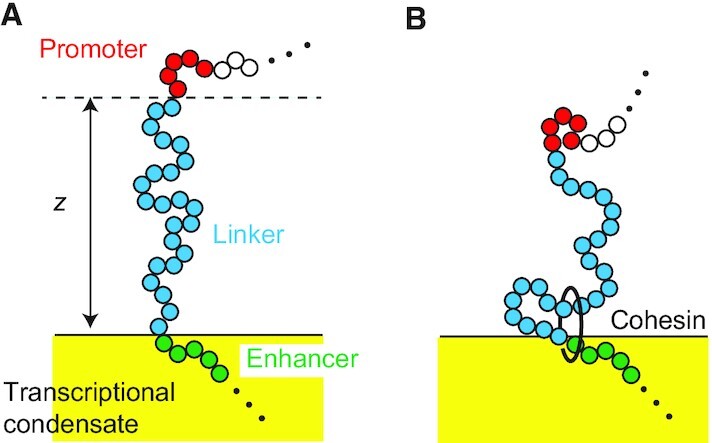
(**A**) Model of a linker chromatin with *N*_0_ units between an enhancer (green) and the promoter (red) of its target gene at the surface of a transcriptional condensate. The enhancer is a part of the super-enhancer and is localized at the surface of a transcriptional condensate. The linker chromatin (blue) between the enhancer and the promoter is expelled from the condensate. (**B**) Cohesin is loaded from the enhancer and translocates chromatin units from the arm region (which has not been extruded) to the loop region (which has been extruded) with a constant rate $\tau _{\rm m}^{-1}$. $z$ is the distance between the position of the promoter and the surface of the condensate.

We consider a linker chromatin between a promoter and an enhancer, which is part of a super-enhancer associated to the surface of a transcriptional condensate, see Figure 1A. We treat a stable transcriptional condensate (>300 nm) (25). The promoter and enhancer have affinities to the transcriptional condensate because of the stochastic binding of transcription factors, whereas the linker chromatin between the promoter and the enhancer is expelled from the condensate (26–28). The linker chromatin is composed of *N*_0_ chain units, Kuhn units, and the promoter is composed of *N*_s_ units, where the length of each unit, the Kuhn length, is *b* for both the linker and the promoter (see also the discussion in the next paragraph). For simplicity, we treat cases in which the number of units in the promoter is smaller than the number of units in the linker chromatin, *N*_s_ ≪ *N*_0_. We also neglect the unbinding of enhancers from the condensate.

The Kuhn length *b* is twice the persistence lengths, with which the correlation function of tangent vectors along the chain decays (as predicted by the worm-like chain model) (33). A Kuhn unit typically contains multiple nucleosomes intervened by stretches of bare DNA (such bare DNA can be called ‘linker DNA’, but we do not use this terminology to avoid confusion with the linker chromatin between the promoter and the enhancer). Recent experiments on yeast showed that the Kuhn length of chromatin is ≈50 nm and there are ≈2 nucleosomes per 10 nm (34). DNA of 146 bps is wound around a histone octamer in each nucleosome and two nucleosomes are intervened by bare DNA of length ≈19 bp; DNA of length 1.65 kb is included in a chain unit. We note that the Kuhn length of chromatin depends on the polynucleosome structure along chromatin and its estimate is rather diverse (34–37), see ref. (38) for a systematic analytical study on the effect of nucleosome spacing on the Kuhn length. Our model neglects the twisting rigidity of chromatin (34,39,40).

In equilibrium, the distribution of the position $z$ of the promoter above the surface of the condensate has the form (41–45)

(1) $$\begin{eqnarray*} \psi _{\rm eq}(z) = \frac{4}{\sqrt{\pi }} \frac{z^2}{(2 l_{\rm eq}^2)^{3/2}} {\rm e}^{- z^2/(2 l_{\rm eq}^2)} \end{eqnarray*}$$

where the mean square end-to-end vector 3*l*^2^ of the linker chromatin is given by

(2) $$\begin{eqnarray*} l_{\rm eq}^2 = \frac{N_0 b^2}{3}. \end{eqnarray*}$$

For simplicity, we analyze only the $z$-position of the promoter, i.e. the position normal to the surface. The factor $z$^2^ in Equation (1) results from the repulsive interactions between the linker chromatin and the transcriptional condensate and from the fact that the linker is part of a longer chromatin chain. The derivation of Equation (1) is shown in the supplementary material (and see also refs. (41–45)). The length scale *l*_eq_ of chromatin of 100 kb length is estimated to be 225 nm for cases in which the Kuhn length *b* is 50 nm and DNA of length 1.65 kb is included in a chain unit.

Following refs. (42–44) we treat the linker chromatin between the promoter and the enhancer as a dumbbell in an effective potential

(3) $$\begin{eqnarray*} U_{\rm eff}(z) = - k_{\rm B}T \log \psi _{\rm eq}(z), \end{eqnarray*}$$

where *k*_B_ is the Boltzmann constant and *T* is the absolute temperature. For cases in which the loop extrusion does not influence the polymer dynamics, the probability distribution function $z$$z$, *t*) of the position $z$ of the promoter at time *t* is derived by the Smoluchowski equation

(4) $$\begin{eqnarray*} \frac{\partial }{\partial t} \psi (z,t) = \frac{k_{\rm B}T}{N_0 \zeta _0} \frac{\partial }{\partial z} \left[ \frac{\partial }{\partial z} \psi (z,t) + \frac{\partial }{\partial z} \left( \frac{U_{\rm eff}(z)}{k_{\rm B}T} \right) \psi (z,t) \right],\nonumber\\ \end{eqnarray*}$$

where *ζ*_0_ is the friction constant of each chromatin unit. The first term of Equation (4) represents the thermal fluctuations (diffusion) of the linker chromatin. The second term of Equation (4) represents the fact that the linker chromatin plays a role in the (entropic) spring of stiffness 3*k*_B_*T*/(*N*_0_*b*^2^) and the contribution of the effective potential −2*k*_B_*T*log $z$ due to the repulsive interactions between the linker chromatin and the surface. The solution of Equation (4) can be derived by using the eigen function expansion, see Section S2 in the Supplementary File.

### Chromatin dynamics during loop extrusion and relaxation

Cohesin is preferentially loaded at the site, at which NIPBL-MAU2 is localized (13–15). Indeed, experiments suggest that the loop extrusion process is driven by the complex of cohesin and NIPBL-MAU2 (19). Recent experiments showed that TADs are recovered relatively fast at superenhancers, implying that there are active loading sites at super-enhancers (21). The loop extrusion may become asymmetric when the cohesin loading site is at the proximity to the elements that stop the loop extrusion (46), such as mediators in the transcriptional condensate (11,12). Motivated by the latter experiments, we treat cases in which cohesin is loaded at the enhancer end of the linker chromatin with a constant rate $\tau _{\rm on}^{-1}$. The loop extrusion by cohesin divides the linker chromatin into the loop and arm regions where only the former region has already been extruded by cohesin, see Figure 1B. Cohesin translocates chromatin units from the arm region to the loop region with a constant rate $\tau _{\rm m}^{-1}$.

**Figure 2.**
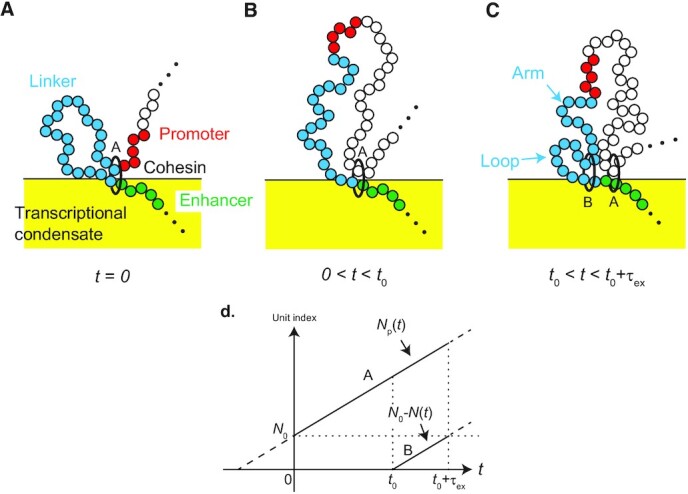
We set *t* = 0 to the time when the gene promoter is translocated to a loop by a cohesin (cohesin A). (**A**) The linker chromatin between the promoter and the enhancer relaxes to the (local) equilibrium conformation in the relaxation process, 0 < *t* < *t*_0_ (**B**). A new cohesin (cohesin B) is loaded at the enhancer and translocates chromatin units from the arm region (which has not been extruded by the new cohesin) to the loop region (which has been extruded by the cohesin) with a constant rate $\tau _{\rm m}^{-1}$ (**C**). The indexes of chromatin units (counted from the enhancer end of the linker chromatin), which are embraced by cohesin A and B, are shown as functions of time *t* (**D**).

For simplicity, we assume that a new cohesin starts the loop extrusion process before the old one is unloaded from the chromatin at the end of the TAD but that there is not more than one cohesin in the linker chromatin between the enhancer and the promoter. A cohesin (cohesin A in Figure 2A) is loaded at the enhancer end of the linker chromatin (at *t* = −*N*_0_*τ*_m_) and translocates the linker chromatin from the arm region to the loop region with a constant rate $\tau _{\rm m}^{-1}$. The loop extrusion is asymmetric because of the mediators bound to the enhancer. We set the time at which the cohesin extrudes the entire linker chromatin to the loop region to *t* = 0, see Figure 2A. At *t* = 0, the promoter is located at the surface of the condensate, ψ($z$, 0) = δ($z$). The number of chromatin units in the loop region increases with time *t* as

(5) $$\begin{eqnarray*} N_{\rm p}(t) = N_0 \left( 1 + \frac{t}{\tau _{\rm ex}} \right), \end{eqnarray*}$$

where *τ*_ex_ ( = *N*_0_*τ*_m_) is the time scale with which a chromatin section of length *N*_0_ is extruded. The linker chromatin is relaxed toward equilibrium during 0 < *t* < *t*_0_ (which we call the relaxation process). We note here that although the size of the loop continues to increase with time *t*, the size of the linker chromatin saturates to the equilibrium value *l*_eq_ when the time period *t*_0_ is longer than the relaxation time, which is necessary for the linker chromatin to return to the equilibrium conformation (given later in Equation (16)) because the linker chromatin is a subsection composed of a fixed number *N*_0_ of units.

A new cohesin (cohesin B in Figure 2C) is loaded at the enhancer end of the linker chromatin and starts the new round of the loop extrusion process at *t* = *t*_0_ (*t*′ = 0). We denote the time elapsed from the loading of the new cohesin as *t*′. The loading of cohesin may be modeled by the Poisson process of rate $\tau _{\rm on}^{-1}$. However, in the following, we assume *t*_0_ = *τ*_on_ to simplify the model, *τ*_ex_ < *τ*_on_; this assumption simplifies the treatment of the loop extrusion process and highlights the roles played by the dynamics of the linker chromatin. The new cohesin produces a new loop region and translocates units in the linker chromatin to this region with a constant rate $\tau _{\rm m}^{-1}$ for *t*_0_ < *t* < *t*_0_ + *τ*_ex_ (0 < *t*′ < *τ*_ex_), which we call loop extrusion process, see Figure 2C. During the loop extrusion process, 0 < *t*′ < *τ*_ex_, the number *N*(*t*) of chromatin units in the arm region is given by

(6) $$\begin{eqnarray*} N(t^{\prime }) = N_0 \left( 1 - \frac{t^{\prime }}{\tau _{\rm ex}} \right), \end{eqnarray*}$$

which is the effective number of chain units between the enhancer and the promoter (the effective number of units in the relaxation process, 0 < *t* < *t*_0_, is *N*(*t*) = *N*_0_). The entire linker chromatin is included in the new loop and the system returns to the initial state at *t* = *t*_0_ + *τ*_ex_. Once the entire linker chromatin is included in the new loop, the loop extrusion by the old cohesin (cohesin A in Figure 2C) does not affect the dynamics of the linker chromatin.

The probability distribution function *ψ*_loc_($z$, *t*) in the local equilibrium has the form

(7) $$\begin{eqnarray*} \psi _{\rm loc}(z,t) = \frac{4}{\sqrt{\pi }} \frac{z^2}{(2 l^2(t))^{3/2}} {\rm e}^{- z^2/(2 l^2(t))}. \end{eqnarray*}$$

The mean square end-to-end distance 3*l*^2^(*t*) of the linker chromatin at the local equilibrium has the form

(8) $$\begin{eqnarray*} l^2(t) = \frac{N(t) (N_{\rm p}(t) - N(t))}{3 N_{\rm p}(t)} b^2, \end{eqnarray*}$$

see Section S1 in the Supplementary File for the derivation. Equation (7) leads to the effective potential

(9) $$\begin{eqnarray*} U_{\rm eff}(z,t) = - k_{\rm B}T \log \psi _{\rm loc}(z,t) \end{eqnarray*}$$

in the relaxation and loop extrusion processes.

During the relaxation process, the time evolution of the probability distribution function $z$$z$, *t*) of the position $z$ of the promoter has the form

(10) $$\begin{eqnarray*}&& \frac{\partial }{\partial t} \psi (z,t;N,N_{\rm p}) + \frac{1}{\tau _{\rm m}} \frac{\partial }{\partial N_{\rm p}} \psi (z,t;N,N_{\rm p}) \nonumber \\ &=& \frac{k_{\rm B}T}{N_0 \zeta _0} \frac{\partial }{\partial z} \left[ \frac{\partial }{\partial z} \psi (z,t;N,N_{\rm p}) + \frac{\partial }{\partial z} \left( \frac{U_{\rm eff}(z,t)}{k_{\rm B}T} \right) \psi (z,t;N,N_{\rm p}) \right]. \nonumber \\ \end{eqnarray*}$$

Equation (10) takes into account the growth of the loop that includes the linker chromatin (see Figure 2B) in an extension of Equation (4), see the second term on the left side of Equation (10). During the loop extrusion process, the time evolution of the probability distribution function $z$$z$, *t*) of the position $z$ of the promoter has the form

(11) $$\begin{eqnarray*} && \frac{\partial }{\partial t} \psi (z,t;N,N_{\rm p}) - \frac{1}{\tau _{\rm m}} \frac{\partial }{\partial N} \psi (z,t;N,N_{\rm p}) + \frac{1}{\tau _{\rm m}} \frac{\partial }{\partial N_{\rm p}} \psi (z,t;N,N_{\rm p}) \nonumber \\ && = \frac{k_{\rm B}T}{N_0 \zeta _0} \frac{\partial }{\partial z} \left[ \frac{\partial }{\partial z} \psi (z,t;N,N_{\rm p}) + \frac{\partial }{\partial z} \left( \frac{U_{\rm eff}(z,t)}{k_{\rm B}T} \right) \psi (z,t;N,N_{\rm p}) \right]. \nonumber \\ \end{eqnarray*}$$

Equation (11) takes into account the extrusion of chromatin units in the arm region to the loop region (see Figure 2C) in an extension of Equation (10), see the third term on the left side of Equation (11).

### Stochastic binding dynamics of promoter to condensate

The fraction σ(*t*) of promoters that bind to the surface of the transcriptional condensate has the form

(12) $$\begin{eqnarray*} \frac{d}{dt} \sigma (t) = k_0 (1 - \sigma (t)) \Psi _a - k_0 \langle {\rm e}^{- n \epsilon /(k_{\rm B}T)} \rangle _n \sigma (t). \end{eqnarray*}$$

**Figure 3.**
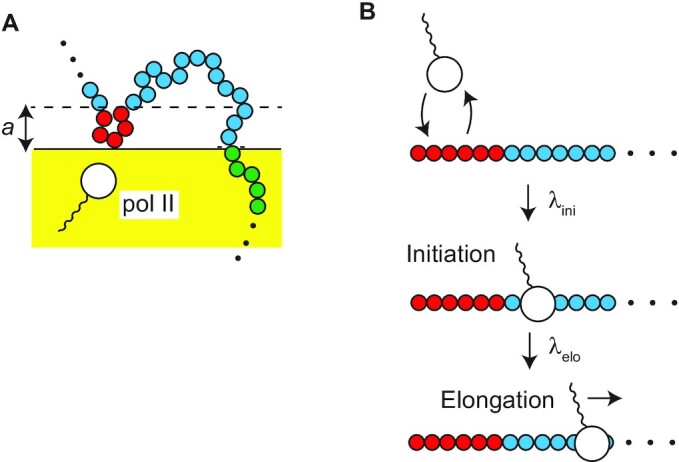
Stochastic binding-unbinding dynamics of promoters at the surface of the transcriptional condensate (**A**). The promoter at the reaction zone 0 < $z$ < *a* binds to the surface of the condensate with a constant rate, where *a* is the size of the promoter. RNA polymerase II (Pol II) in the condensate binds to and unbinds from the promoter (**B**). The equilibrium constant *K*_ini_ accounts for the binding-unbinding dynamics. The bound Pol II starts transcription and shows the promoter proximal pause (the initiation state). The rate constant *λ*_ini_ accounts for the transition to this state. The promoter is tethered to the surface of the condensate by Pol II in the initiation state. Pol II escapes with the rate constant *λ*_elo_ from the promoter to start elongation.

The first term is the binding rate of the promoter to the condensate and the second term is the unbinding rate of the promoter from the condensate. *k*_0_ is the rate constant that accounts for this process. We assume that the promoter binds to the surface of the condensate with a constant rate *k*_0_ when it is located at the reaction zone 0 < $z$ < *a* due to the finite size of the promoter, see Figure 3A. *Ψ*_*a*_ is the probability with which the promoter is located at the reaction zone

(13) $$\begin{eqnarray*} \Psi _a = \lim _{\tau \rightarrow \infty } \frac{1}{\tau } \int _0^\tau dt \, \int _0^a dz \, \psi (z,t). \end{eqnarray*}$$

Experiments by Nozaki and coworkers suggest that Pol II molecules in the initiation state tether the promoter, to which these molecules bind, to the condensate (47). The Boltzmann factor $\langle {\rm e}^{- n \epsilon /(k_{\rm B}T)} \rangle _n$ accounts for the tethering of the promoter to the condensate by Pol II. *ε* is the binding energy between the condensate and Pol II bound to the promoter. 〈〉_*n*_ is the average with respect to the number *n* of Pol II bound to the promoter (in the initiation state, see Figure 3B and the discussion below). Equation (12) therefore takes into account the chromatin dynamics and the tethering of the promoter by Pol II in the initiation state in an extension of the Langmuir’s theory of the dynamics of surface adsorption (48). With Equation (12), we assume that the binding of the gene promoter to the transcriptional condensate is rate limited, motivated by the fact that among the genes that are at the proximity to a condensate and move together with the condensate, only $20\%$ of them colocalize with the condensate (25), see also the Discussion.

We simplify a transcription model used by Stasevich and coworkers (50) to derive the form of the factor $\langle {\rm e}^{- n \epsilon /(k_{\rm B}T)} \rangle _n$. Pol II shows stochastic binding and unbinding dynamics to the promoter, see Figure 3B. When Pol II bound to the promoter changes its conformation and assembles the preinitiation complex, the enzyme starts transcription and stops ∼100 nucleotides downstream of the transcription starting site (49). The pausing state of Pol II is called the initiation state. Pol II then starts elongation when its carbon terminal domain is phospholylated (49). The time evolution equation for the probability *P*_ini_(*t*) that the promoter is occupied by Pol II in the initiation state has the form

(14) $$\begin{eqnarray*} \frac{d}{dt} P_{\rm ini}(t) = \lambda _{\rm ini}\frac{\rho }{\rho + K_{\rm ini}} (1 - P_{\rm ini}(t)) - \lambda _{\rm elo}P_{\rm ini}(t). \end{eqnarray*}$$

The first term of Equation (14) is the rate with which Pol II enters the initiation state and the second term is the rate with which Pol II enters elongation state. *ρ* is the concentration of Pol II in the transcriptional condensate and *K*_ini_ is the equilibrium constant with respect to the stochastic binding and unbinding dynamics of Pol II to the promoter. *λ*_ini_ denotes the rate with which Pol II bound to the promoter becomes the initiation state. *λ*_elo_ is the rate with which Pol II in the initiation state enters the elongation state. For simplicity, we treat cases in which not more than one Pol II can occupy a promoter. We also assume that the difference of Pol II concentration between the interior and exterior of the transcriptional condensate is very large and neglect the transcription by Pol II in the exterior of the transcriptional condensate. We use the probability *P*_ini_(*t*) in the steady state to derive the factor $\langle {\rm e}^{- n \epsilon /(k_{\rm B}T)} \rangle _n$, assuming that the transcription dynamics is faster than the binding-unbinding dynamics of the promoter to the condensate.

## RESULTS

### Relaxation time of linker chromatin increases as length of linker chromatin increases

To understand the chromatin dynamics during the loop extrusion process and during the relaxation process, we first analyze the distribution function $z$$z$, *t*) of the position of the promoter. When the promoter is extruded by a cohesin, the promoter is located at the surface of the condensate $z$$z$, 0) = $z$$z$). The linker chromatin between the promoter and the enhancer is now included in one loop. The linker chromatin shows the relaxation dynamics towards the equilibrium conformation, while the number of units in the chromatin loop increases with time due to the loop extrusion. As a result the promoter diffuses away from the surface with time due to the thermal fluctuation of the linker chromatin, see Figure 5A. The distribution function eventually becomes that of the equilibrium, if the linker chromatin is completely relaxed before new cohesin is loaded to the linker chromatin, see Figure 5B.

**Figure 4.**
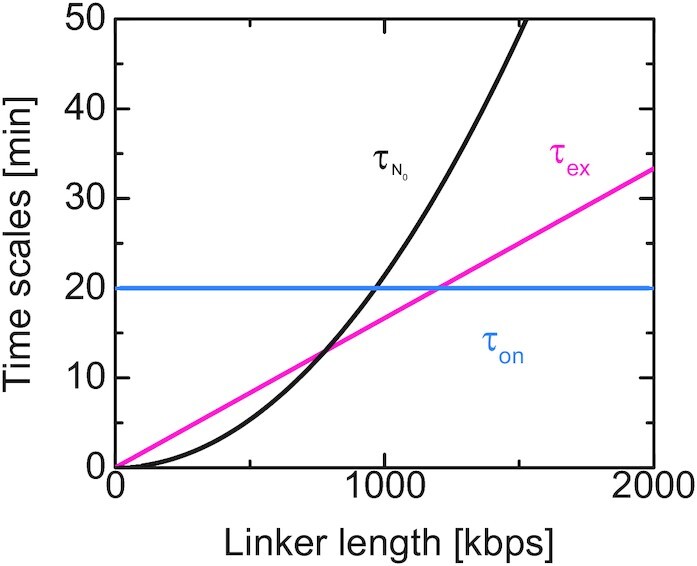
Estimates of the relaxation time $\tau _{N_0}(= N_0^2 \tau _1)$ (black), the time scale *τ*_ex_ ( = *N*_0_*τ*_m_) to extrude the linker chromatin (magenta), and the loading time *τ*_on_ of cohesin (cyan). These estimates correspond to the case in which the length *b* of a chain unit is 50 nm and 10 nucleosomes is included in it (34). A nucleosome is composed of DNA of length 146 bps wound around a histone octamer and nucleosomes are linked by DNA of length 19 bp; DNA of 1.65 kb is included in a chain unit. We estimated *τ*_1_ ≈ 0.0035 s from the mean square displacement of nucleosomes in HeLa cells ($8b^2/(3 \pi ^{3/2}\sqrt{\tau _1}) \approx 0.02{\rm \mu m^2/s^{1/2}}$) (47). The time scale of loop extrusion is estimated by using the fact that the average extrusion rate of cohesin in HeLa cells is 1 kpbs/s (19). The time scale of the recovery of topologically associated domains is 20–40 min in a human colorectal carcinoma cell line (21). If the dynamics of assembling the topologically associated domains is limited by the loading of cohesin, the loading time τ_on_ is a value of the same order. In these estimates, we used quantities determined by different experiments on different organisms and by no means absolute. Estimates of the time scales for chain units, *τ*_1_ and *τ*_m_, are shown as functions of the unit length *b* in Supplementary Figure S1 in the Supplementary File.

**Figure 5.**
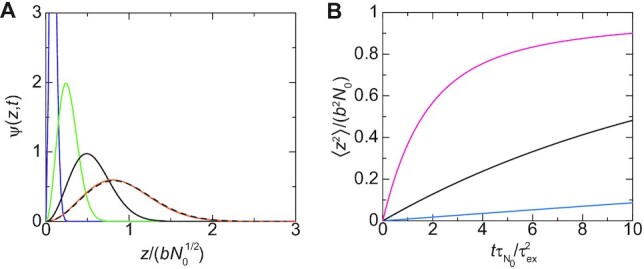
(**A**) The distribution function ψ($z$, *t*) of the promoter in the relaxation process is shown as a function of the position $z$ of the promoter for $t \tau _{N_0}/\tau _{\rm ex}^2 = 1.0$ (purple), 10.0 (light green), 50.0 (black), and 500.0 (orange). We used $\tau _{\rm ex}/\tau _{N_0}= 0.1$. The broken curve is the distribution function at equilibrium. **(B)** The mean square distance between the promoter and the surface of the condensate is shown as a function of rescaled time $t \tau _{N_0}/\tau _{\rm ex}^2$ for $\tau _{\rm ex}/\tau _{N_0}= 0.1$ (cyan), 0.3 (black) and 1.0 (magenta), where the ratio $\tau _{\rm ex}/\tau _{N_0}$ is proportional to the inverse of the number *N*_0_ of units in the linker chromatin. The rescaling factor $\tau _{\rm ex}^2/\tau _{N_0}$ does not depend on the number *N*_0_ of units in the linker chromatin.

The distribution function of the promoter has the form

(15) $$\begin{eqnarray*} \psi (z,t) = \frac{4}{\sqrt{\pi }} \frac{z^2}{(2 l_{\rm eq}^2 r(t))^{3/2}} {\rm e}^{- z^2/(2 l_{\rm eq}^2 r(t))} \end{eqnarray*}$$

for both the relaxation process and the loop extrusion process, where $3 l_{\rm eq}^2(= N_0 b^2)$ is the mean square distance between the promoter and the enhancer in the equilibrium distribution. The relaxation factor *r*(*t*) is proportional to the mean square distance between the promoter and the enhancer, 〈$z$^2^〉 = *b*^2^*N*_0_*r*(*t*), and its form depends on the process, see Equation (S47) in the Supplementary File for the form of *r*(*t*) in the relaxation process. The relaxation becomes slower as the number *N*_0_ of units in the linker chromatin increases, see Figure 5B. The relaxation time, which is necessary for the linker chromatin to return to the equilibrium conformation, has the form

(16) $$\begin{eqnarray*} \tau _{N_0}= \frac{N_0^2 \zeta b^2}{6 k_{\rm B}T}, \end{eqnarray*}$$

which increases with increasing the number *N*_0_ of units in the linker chromatin, see Figure 4 for an estimate. The fact that the relaxation time $\tau _{N_0}$ is proportional to the square of the number *N*_0_ of units reflects the Rouse dynamics of the linker chromatin (31).

**Figure 6.**
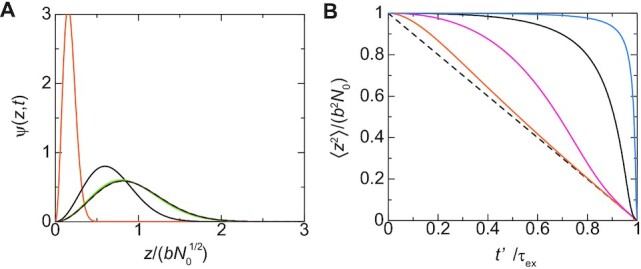
**(A)** The distribution function ψ($z$, *t*) is shown as a function of the position $z/(bN_0^{1/2})$ of the promoter for *t*′/*τ*_ex_ = 0.0 (black broken curve), 0.3 (brown), 0.6 (light green), 0.9 (black), and 0.98 (orange), where *t*′ ( = *t* − *t*_0_) is the time elapsed since the loop extrusion process starts. We used $\tau _{\rm ex}/\tau _{N_0}= 0.1$. **(B)** The mean square distance between the promoter and the surface is shown as a function of time *t*′/*τ*_ex_ for $\tau _{\rm ex}/\tau _{N_0}= 0.01$ (cyan), 0.1 (black), 1.0 (magenta), and 10.0 (orange). The linker chromatin is completely relaxed (*t*_0_ → ∞) when the loop extrusion starts for both (**A**) and (**B**).

A new cohesin is loaded at the enhancer end of the linker chromatin and a new loop extrusion process starts at time *t*_0_. The new cohesin translocates the chromatin units in the arm region to the loop region and thus the promoter is dragged towards the surface of the condensate, see Figure 6. The distribution function of the promoter has the form of Equation (15), but the form of the relaxation factor *r*(*t*) is different from the relaxation process, see Equation (S56) in the Supplementary File. For cases in which the ratio $\tau _{\rm ex}/\tau _{N_0}$ of time scales is very large, the promoter approaches the surface with almost constant rate, see the orange line in Figure 6B. In contrast, for small values of the ratio $\tau _{\rm ex}/\tau _{N_0}$, the position of the promoter is hardly affected by the loop extrusion process for a finite period of time before the mean square distance between the promoter and the surface decreases steeply, see the cyan line in Figure 6. This results from the fact that the tension generated by the loop extrusion process diffuses along the linker chromatin and it takes a finite time until the tension drags the promoter towards the surface. This feature agrees well with our previous theory based on a bead-spring model (29) and Onsager’s variational principle (30).

**Figure 7.**
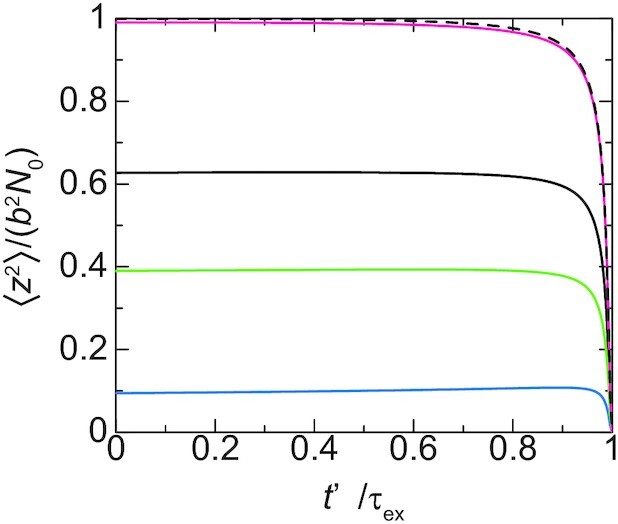
The mean square distance between the promoter and the surface is shown as a function of time *t*′/τ_ex_ elapsed since the loop extrusion process starts at $t_0/\tau _{N_0}= 0.1$ (cyan), 0.5 (light green), 1.0 (black) and 5.0 (magenta). We used $\tau _{\rm ex}/\tau _{N_0}= 0.01$. The black broken curve is calculated for an asymptotic limit, *t*_0_ → ∞.

For cases in which the time *t*_0_ at which a new cohesin is loaded is larger than the relaxation time $\tau _{N_0}$ of the linker chromatin, the linker chromatin is relaxed to the equilibrium conformation before the loop extrusion process starts, see the magenta line in Figure 7. In contrast, for cases in which the time period *t*_0_ is shorter than the relaxation time $\tau _{N_0}$, the loop extrusion process starts before the linker chromatin is completely relaxed to the equilibrium conformation, see the cyan, light green and black lines in Figure 7. In the latter case, the promoter thus stays in the proximity of the surface because it is dragged to the surface by the loop extrusion that happens with a constant rate $\tau _{\rm on}^{-1}$.

Our theory therefore predicts that the average loading time *τ*_on_ of cohesin and the relaxation time $\tau _{N_0}$ of the linker chromatin are the important time scales that determine the distribution of the position of the promoter.

### Contact probability of promoters to transcriptional condensate

The solution of Equation (14) for the steady state predicts that the factor $\langle {\rm e}^{- n \epsilon /(k_{\rm B}T)} \rangle _n$ is derived as

(17) $$\begin{eqnarray*} \langle {\rm e}^{- n \epsilon /(k_{\rm B}T)} \rangle _n = \frac{\alpha _{\rm elo}\rho + K_{\rm elo}}{\rho + K_{\rm elo}}, \end{eqnarray*}$$

where *K*_elo_ ( = *λ*_elo_*K*_ini_/(*λ*_elo_ + *λ*_ini_)) is the effective equilibrium constant. The factor *α*_elo_ has the form

(18) $$\begin{eqnarray*} \alpha _{\rm elo}= \frac{\lambda _{\rm elo}}{\lambda _{\rm elo}+ \lambda _{\rm ini}} + \frac{\lambda _{\rm ini}}{\lambda _{\rm elo}+ \lambda _{\rm ini}} {\rm e}^{- \epsilon /(k_{\rm B}T)}. \end{eqnarray*}$$

Equation (17) has the asymptotic form $\langle {\rm e}^{- n \epsilon /(k_{\rm B}T)} \rangle _n = {\rm e}^{- \epsilon /(k_{\rm B}T)}$ for small rate constant *λ*_elo_ (because the promoter is occupied by pol II in the initiation state most of time) and $\langle {\rm e}^{- n \epsilon /(k_{\rm B}T)} \rangle _n = 1$ for large rate constant *λ*_elo_ (because the promoter is not occupied by pol II in the initiation state most of time).

**Figure 8.**
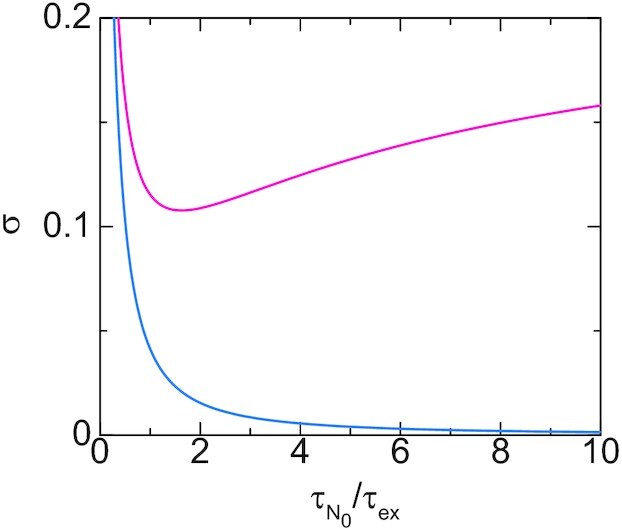
The contact probability of a promoter to the surface of the transcriptional condensate is shown as a function of the ratio $\tau _{N_0}/\tau _{\rm ex}$ of time scales for cases in which the loop extrusion is active (magenta) and not active (cyan). We used *α*_elo_ = 0.3 and *ρ*/*K*_elo_ = 2.2 for the calculations. For the magenta line we set $t_0 \tau _{N_0}/\tau _{\rm ex}^2 = 10.0$, 2*k*_B_*Tτ*_m_/(*ζ*_0_*a*^2^) = 5.0 (the cyan line corresponds to $t_0 \tau _{N_0}/\tau _{\rm ex}^2 \rightarrow \infty$). The ratio $\tau _{N_0}/\tau _{\rm ex}$ scales linear to the number *N*_0_ of units in the linker chromatin, whereas the time scale $\tau _{N_0}/\tau _{\rm ex}^2$ does not depend on the number *N*_0_ of units.

In this section, we treat cases in which the concentration *ρ* of Pol II in the condensate is constant because the number of genes anchored to the condensate is small and the concentration *ρ* is relaxed to the equilibrium value in a relatively short time. For cases in which the loop extrusion is inhibited, the contact probability *σ* decreases monotonically as the number *N*_0_ of units in the linker chromatin increases, see the cyan line in Figure 8 (the ratio $\tau _{N_0}/\tau _{\rm ex}$ of time scales is proportional to the number *N*_0_ of units). This is expected from the polymer physics: the linker chromatin acts an entropic spring which anchors the promoter to the surface of the transcription condensate and the stiffness of spring decreases as the number *N*_0_ of units in the linker chromatin increases (31,32). In sharp contrast, for cases in which the loop extrusion is active, the contact probability *σ* is a non-monotonic function of the number *N*_0_ of units in the linker chromatin, see the magenta line in Figure 8. In fact, the contact probability *σ* increases with the number *N*_0_ of units (for large enough *N*_0_). This is because the loop extrusion process drags the promoter to the surface with a constant rate and the relaxation time $\tau _{N_0}$, by which the linker chromatin returns to the equilibrium conformation, increases as the number *N*_0_ of units in the linker chromatin increases, see Equation (16) and Figure 4: the promoter stays at the proximity to the surface for longer time as the number *N*_0_ of units in the linker chromatin increases. On the other hand, the contact probability *σ* approaches asymptotically to the equilibrium values as the number *N*_0_ of segment decreases.

**Figure 9.**
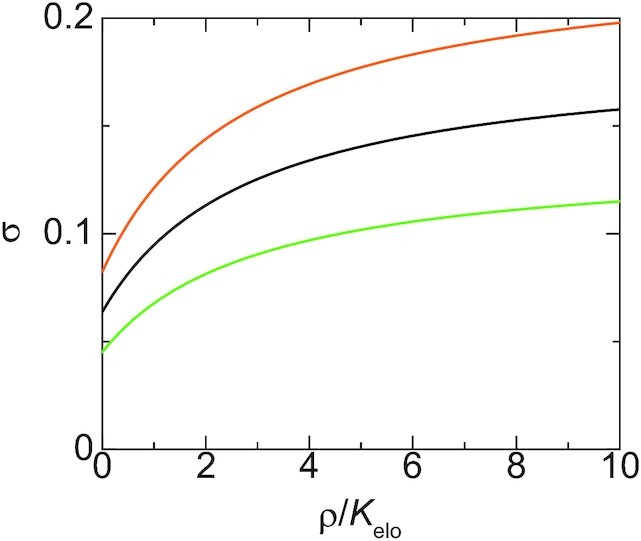
The contact probability of a promoter to the surface of the transcriptional condensate is shown as a function of the concentration *ρ* of Pol II in the condensate for $\tau _{N_0}/\tau _{\rm ex}= 1.0$ (light green), 10.0 (black), 20.0 (orange). We used *α*_elo_ = 0.3, $t_0 \tau _{N_0}/\tau _{\rm ex}^2 = 20.0$, 2*k*_B_*Tτ*_m_/(*ζ*_0_*a*^2^) = 5.0 for the calculations.

The contact probability *σ* is represented as

(19) $$\begin{eqnarray*} \sigma = \frac{\Psi _a (\rho + K_{\rm elo})}{(\Psi _a + \alpha _{\rm elo}) \rho + (1 + \Psi _a ) K_{\rm elo}} \end{eqnarray*}$$

by using Equation (17), see Figure 9. The contact probability σ thus increases as the concentration *ρ* of Pol II increases (*α*_elo_ < 1 for *ε* > 0, see Equation (18), and the factor *Ψ*_*a*_ does not depend on the concentration *ρ* of Pol II). The contact probability has an asymptotic form *σ* = *Ψ*_*a*_/(*Ψ*_*a*_ + *α*_elo_) for large concentrations *ρ*. The asymptotic probability reaches unity only for *λ*_elo_/*λ*_ini_ → 0 because Pol II tethers the promoter to the transcriptional condensate only in the initiation state. Our theory therefore predicts that the concentration of pol II in the condensate and the elongation rate are also important factors that determine the contact probability of the gene promoter to the transcriptional condensate.

## DISCUSSION

Our theory predicts the contact probability of a promoter of a gene to the surface of a transcriptional condensate when the gene is anchored to the condensate via a superenhancer. This theory treats a relatively large transcriptional condensate, which is stable for the experimental time scale (25). Transcription machineries in the condensate are available to the promoter of the gene when the promoter is bound to the condensate. The contact probability therefore corresponds to the ratio of time in which the transcription of the gene is active during the transcription bursting. For cases in which the loop extrusion is inhibited, the contact probability decreases as the number of chromatin units in the linker chromatin increases. This reflects the fact that the linker chromatin acts as an entropic spring which anchors the promoter to the surface of the condensate and the stiffness of the spring decreases as the number of chromatin units in the linker chromatin increases. In contrast, for cases in which the loop extrusion is active, the contact probability of the promoter to the transcriptional condensate increases as the number of chromatin units in the linker chromatin increases as long as the promoter and the enhancer are in the same TAD. This is because the loop extrusion process drags the promoter to the surface with a constant rate and the relaxation time with which the promoter stays at the proximity to the surface increases as the number of chromatin units in the linker chromatin increases. This situation is very different from the static loops assumed in other theories (8,9).

We used a couple of assumptions to simplify the theory:

1. The binding and unbinding dynamics of the gene promoters to the condensate is rate limited. It is motivated by the fact that among the genes that are at the proximity to a condensate and move together with the condensate, only $20\%$ of them colocalize with the condensate (25). If the binding of the gene promoters is diffusion limited, the unbinding of these promoters is a rare event: most of gene promoters would bind to the condensate in the steady state (or the transcriptional condensate observed in (25) is in the transient state). The limiting process may also depend on the transcription factors that bind to the gene promoter.
2. Cohesin is trapped at the surface of the condensate. This is probably the case because the loop extrusion starts from the (super)enhancer (21) and mediators may act as ‘boundary elements’ that stop the loop extrusion by cohesin (11,12). Single molecule experiments revealed that cohesin shows symmetric loop extrusion when there are no boundary elements (19). However, Hi-C experiments showed the signature of asymmetric loop extrusion, probably because cohesin loading sites are in the proximity to boundary elements in such cases (46). Indeed, our main prediction reflects the dynamics of the linker chromatin in the relaxation process and is not very sensitive to the details of the dynamics in the loop extrusion process, as long as the average loading time *τ*_on_ is larger than the time scale *τ*_ex_ of the loop extrusion process.
3. The linker chromatin shows repulsive interactions with the transcriptional condensate. This treatment is motivated by the fact that chromatin tends to be excluded from the transcription condensate (26–28).
4. There are at most one cohesin in the linker chromatin. The cases in which there are multiple cohesin molecules in the linker chromatin are treated by using *N*_p_ = *N*_0_*τ*_on_/*τ*_ex_ and *N*(*t*) = *N*_0_(*τ*_on_ − *t*)/*τ*_ex_.
5. We treated a relatively large condensate, which is stable for the experimental time scale (with which mouse embryonic stem cells are differentiated) (25). The lifetime of small transcriptional condensates is in the order of 10 s. The assembly and disassembly of such condensates may be coupled with processes involved in the transcription dynamics, such as the phospholylation of the C terminal domain of pol II (51) and synthesis of RNA (52). We also assumed that the concentration of pol II in the condensate does not depend on the contact probability of gene promoters. Pol II with hyper-phospholylated C terminal domains does not have affinity to transcriptional condensates (51). The pol II in the condensate is thus determined by the transcription rate and the relaxation time with which the concentration of pol II in the condensate relaxes to the equilibrium concentration. We assumed that the relaxation time with respect to the concentration of pol II is small, relative to the transcription rate, which is probably the cases in large transcriptional condensates (25).
6. The contact probability of a promoter to the condensate is not affected by other gene promoters.
7. We neglected the excluded volume interactions, the hydrodynamic interactions, and the topological interactions between chromatin chains. Indeed, recent experiments on He-La cells (47) revealed that nucleosomes show subdiffusion that is characteristic to the polymer dynamics without these interactions. The intra-chain excluded volume interactions are screened by the inter-chain excluded volume interactions and the hydrodynamic interactions are screened by the friction between the surrounding chains and the solvent; both of these interactions are negligible in a concentrated polymer solution (31). In the context of the linker chromatin at the surface of a transcriptional condensate, the contributions of the excluded volume interactions and hydrodynamic interactions depend on the density of chromatin chains, which are anchored to the condensate (30) and the concentration of chromatin in the nucleoplasm. The topological interactions between chains are significant for length scales larger than the tube diameter (31), but may not be significant in the chromatin dynamics due to the action of topoisomerase IIA (11,53,54).
  The chromatin dynamics may also depend on the organism and the stage of development. Indeed, recent experiments suggest that the chromatin dynamics is affected by intra-chromosome transient bonding in yeast (34) and by topological interactions (55) in the zygote of *C. elegans*. The contributions of the excluded volume interactions, the hydrodynamic interactions, and the topological interactions to the polymer dynamics have been extensively studied (31) and can be taken into account in an extension of our theory.
8. The binding between the promoter and the condensate is enhanced by pol II in the initiation state. This treatment is motivated by the recent finding that transcriptional condensates have affinity with the C terminal domains of pol II, but not when the C terminal domains are hyperphosphorylated (51,56). Pol II stops at the proximity of the promoter in the initiation state, in contrast to the elongation state, where pol II shows the conformational transitions while it moves uni-directionally along DNA. The binding energy ε is the free energy that takes into account the conformational fluctuation of pol II in the initiation state. For simplicity, we represented the enhancement by using a single value ε of the binding (free) energy, see Equation (12). However, the binding energy may depend on the extent of the phosphorylation of the C terminal domains.

It is of interest to theoretically predict (i) the coupling between the assembly/disassembly of transcriptional condensates and (ii) the transcription dynamics and the interaction between gene promoters by using an extension of our theory.

There are growing number of researches on phase separation in biological systems (57). Many researches emphasize the fact that the mutivalent interactions between intrinsically disordered domains of proteins play an important role in the formation of condensates (57) and that specific proteins and RNA are localized in condensates (25). There is another important aspect of phase separation: phase separation creates interfaces. Superenhancers are anchored at the interface between a transcriptional condensate and the nucleoplasm. Our theory predicts that the promoters of the target genes of the superenhancers stay at the proximity to the interface because the loop extrusion process draggs the promoters to the interface and the slow dynamics of the linker chromatin between the promoter and the enhancer. Interfaces are asymmetric and (quasi-) 2D systems. Elucidating the roles played by interfaces in the biochemical reactions is an interesting avenue of phase separation researches in biological systems.

## DATA AVAILABILITY

The Mathematica file (transcriptiondynamicsVer8.nb) used to derive the data that support the findings of this study are available in figshare with the identifier (https://doi.org/10.6084/m9.figshare.14102156).

## Supplementary Material

gkab275_Supplemental_File
