# Supplementary material for: Slow chromatin dynamics enhances promoter accessibility to transcriptional condensates
Source: Nucleic Acids Res. 2021 Apr 22;49(9):5017–27. doi: 10.1093/nar/gkab275 (PMC8136786; doi:10.1093/nar/gkab275)
Supplement: gkab275_Supplemental_File [file gkab275_supplemental_file.pdf]

Supplementary File: Slow chromatin dynamics enhance promoter accessibility to transcriptional condensates

E-mail:

S1 Probability distribution function at (local) equilibrium

The probability distribution function $\psi_{\rm eq}(z)$ at equilibrium has the form

$$\psi_{\rm eq}(z) = \frac{q(z,N)q^*(z,N)}{\int_0^\infty dz q(z,N)q^*(z,N)}. \tag{S1}$$

The functions $q(z,s)$ and $q^*(z,s)$ are the solutions of the Edwards equation

$$\frac{\partial}{\partial s}q(z,s) = \frac{b^2}{6}\frac{\partial^2}{\partial z^2}q(z,s) \tag{S2}$$

$$\frac{\partial}{\partial s}q^*(z,s) = \frac{b^2}{6}\frac{\partial^2}{\partial z^2}q^*(z,s), \tag{S3}$$

where z is the distance from the surface of the condensate and s is the coordinate along the chromatin chain. b denotes the Kuhn length. Eq. (S1) is the probability distribution function of the N-th unit in a chromatin chain, which is tethered to the surface of the condensate at $s = 0$ and $s = N_{\rm p}$. The boundary conditions for eqs. (S2) and (S3) are therefore

$$q(z,0) = q^*(z,N_{\rm p}) = \delta(z-\epsilon) \tag{S4}$$

$$q(0,s) = q^*(0,s) = 0. \tag{S5}$$

Eqs. (S2) - (S5) imply $q^*(z,s) = q(z,N_{\rm p}-s)$.

By separating the variables $q(z,s) = R(z)S(s)$, eq. (S2) is decomposed to equations for $R(z)$ and $S(s)$

$$\frac{\partial}{\partial s}S(s) = -\lambda S(s) \tag{S6}$$

$$\frac{\partial^2}{\partial z^2}R(z) = -\frac{6\lambda}{b^2}R(z). \tag{S7}$$

The solutions of eqs. (S6) and (S7) has the form

$$S(s) = \mathrm{e}^{-\lambda s} \tag{S8}$$

$$R(z) = \sin(kz) \tag{S9}$$

with

$$\lambda = \frac{k^2 b^2}{6}. \tag{S10}$$

We omitted $\cos(kz)$ from the solution of eq. (S9) by using the boundary condition, eq. (S5). The solution of eq. (S2) has the form

$$\begin{aligned} q(z,s) &= \frac{2}{L}\sum_k \sin(k\epsilon)\sin(kz)\mathrm{e}^{-k^2b^2s/6} \\ &= \frac{1}{\pi}\int_{-\infty}^{\infty} dk\, \sin(k\epsilon)\sin(kz)\mathrm{e}^{-k^2b^2s/6} \\ &= \sqrt{\frac{3}{2\pi b^2 s}}\left(\mathrm{e}^{-3(z-\epsilon)^2/(2b^2s)} - \mathrm{e}^{-3(z+\epsilon)^2/(2b^2s)}\right) \end{aligned} \tag{S11}$$

where we used eq. (S4) to derive the first line of eq. (S11). In the limit $\epsilon \to 0$, eq. (S11) has the asymptotic form

$$q(z,s) = \sqrt{\frac{3}{2\pi b^2 s}}\frac{6\epsilon}{b^2 s} z\mathrm{e}^{-3z^2/(2b^2s)}. \tag{S12}$$

In a similar manner, the function $q^*(z,s)$ is derived in the form

$$q^*(z,s) = \sqrt{\frac{3}{2\pi b^2(N_\mathrm{p}-s)}}\frac{6\epsilon}{b^2(N_\mathrm{p}-s)} z\mathrm{e}^{-3z^2/(2b^2(N_\mathrm{p}-s))}. \tag{S13}$$

The probability distribution function $\psi_\mathrm{eq}(z)$ at equilibrium has the form

$$\psi_\mathrm{eq}(z) = \frac{4}{\sqrt{\pi}}\frac{z^2}{(2l_\mathrm{p}^2)^{3/2}}\mathrm{e}^{-z^2/(2l_\mathrm{p}^2)} \tag{S14}$$

with

$$l_{\mathrm{p}}^2 = \frac{b^2}{3}\frac{N(N_{\mathrm{p}}-N)}{N_{\mathrm{p}}}. \tag{S15}$$

Eq. (S14) is reduced to the probability distribution function of the position z of a promoter in a very long chromatin for $N_{\mathrm{p}} \to \infty$, see eq. (2).

With the loop extrusion process, the number $N_{\mathrm{p}}(t)$ of units in the loop, in which the chromatin section between the promoter and the enhancer is included, increases with time and has the form

$$N_{\mathrm{p}}(t) = N_0\left(1 + \frac{t}{\tau_{\mathrm{ex}}}\right), \tag{S16}$$

where N_0 is the number of units in the linker chromatin between the promoter and the enhancer and τ_{ex} is the time scale, with which the linker chromatin is extruded, see eq. (5) in the main article. The number $N(t)$ of units in the arm region is N_0 in the relaxation process, $0 < t < t_0$, and has the form

$$N(t') = N_0\left(1 - \frac{t'}{\tau_{\mathrm{ex}}}\right), \tag{S17}$$

in the loop extrusion process, $t_0 < t < t_0 + \tau_{\mathrm{ex}}$, see eq. (6) in the main article. t' $(= t - t_0)$ is the time elapsed since the loop extrusion starts. The probability distribution function at the local equilibrium is derived by substituting eqs. (S16) and (S17) into eq. (S15), see eqs. (7) and (8) in the main article.

S2 Probability distribution function in relaxation process

The time evolution of the probability distribution function $\psi(z,t)$ is given by the Smoluchowski equation

$$\frac{\partial}{\partial t}\psi(z,t) = \frac{k_{\mathrm{B}}T}{N\zeta}\frac{\partial}{\partial z}\left[\frac{\partial}{\partial z}\psi(z,t) + \frac{\partial}{\partial z}\left(\frac{U_{\mathrm{eff}}(z)}{k_{\mathrm{B}}T}\right)\psi(z,t)\right], \tag{S18}$$

where $U_{\mathrm{eff}}(z)$ is the effective potential that has the form

$$\frac{U_{\mathrm{eff}}(z)}{k_{\mathrm{B}}T} = \frac{z^2}{2l_{\mathrm{p}}^2} - 2\log z, \tag{S19}$$

see eqs. (1) - (4) in the main article. We derive the solution $\psi(z,t)$ of eq. (S18) in the form

$$\psi(z,t) = \frac{4}{\sqrt{\pi}}\frac{1}{(2l_{\mathrm{p}}^2)^{3/2}}\Phi(z,t)\mathrm{e}^{-U_{\mathrm{eff}}(z)/(k_{\mathrm{B}}T)}. \tag{S20}$$

Substituting eq. (S20) into eq. (S18) leads to the form

$$\frac{\partial}{\partial t}\Phi(z,t) = \frac{k_{\mathrm{B}}T}{N\zeta}\left[\frac{\partial^2}{\partial z^2}\Phi(z,t) - \left(\frac{z}{l_{\mathrm{p}}^2} - \frac{2}{z}\right)\frac{\partial}{\partial z}\Phi(z,t)\right]. \tag{S21}$$

By separating the variables $\Phi(z,t) = Z(z)T(t)$, eq. (S21) is decomposed to equations for $Z(z)$ and $T(t)$

$$\frac{\partial}{\partial t}T(t) = -\omega T(t) \tag{S22}$$

$$\frac{\partial^2}{\partial z^2}Z(z) - \left(\frac{z}{l_{\mathrm{p}}^2} - \frac{2}{z}\right)\frac{\partial}{\partial z}Z(z) = -\frac{N\zeta\omega}{k_{\mathrm{B}}T}Z(z). \tag{S23}$$

With the variable transformation

$$\xi = \frac{z^2}{2l_{\mathrm{p}}^2}, \tag{S24}$$

eq. (S23) is rewritten in the form

$$\xi Z''(\xi) + \left(\frac{3}{2} - \xi\right) Z'(\xi) + \frac{N\zeta l_{\mathrm{p}}^2 \omega}{2k_{\mathrm{B}}T} Z(\xi) = 0. \tag{S25}$$

Eq. (S25) is the Laguerre equation. The solution of eq. (S25) has the form

$$Z_n(\xi) = L_n^{1/2}(\xi) \tag{S26}$$

$$\omega_n = \frac{2k_{\mathrm{B}}T}{N\zeta l_{\mathrm{p}}^2} n, \tag{S27}$$

where $L_n^{1/2}(\xi)$ ($n = 0,\ 1,\ 2,\ \cdots$) is the Laguerre polynomials of the n-th rank. We rewrite $Z(\xi)$ and ω to $Z_n(\xi)$ and ω_n, respectively, because these functions depend on the index n. The solution of eq. (S22) is readily derived as

$$T_n(t) = T_n(0)\mathrm{e}^{-\omega_n t}. \tag{S28}$$

We rewrite $T(t)$ to $T_n(t)$ because this function depends on the index n. The solution of eq. (S21) therefore has the form

$$\Phi(z,t) = \sum_{n=0}^{\infty} C_n(0) L_n^{1/2}(\xi) \mathrm{e}^{-\omega_n t}, \tag{S29}$$

where $C_n(0)$ ($n = 0,\ 1,\ 2,\ \cdots$) are constants that are determined by the initial condition. By substituting eq. (S29) into eq. (S20), the general solution of the probability distribution function $\psi(z,t)$ is derived as

$$\psi(z,t)dz = \sum_{n=0}^{\infty} C_n(0) L_n^{1/2}(\xi) \xi^{1/2} \mathrm{e}^{-\xi} \mathrm{e}^{-\omega_n t} d\xi. \tag{S30}$$

By using the probability distribution function $\psi_0(z)$ ($\equiv \psi(z,0)$) at $t = 0$, the coefficients

$C_n(0)$ are written as

$$C_n(0) = \frac{\Gamma(n+1)}{\Gamma(n+3/2)} \int_0^\infty dz\, L_n^{1/2}(\xi)\psi_0(z). \tag{S31}$$

Eq. (S31) is derived by using the orthogonality of the Laguerre polynomials

$$\int_0^\infty d\xi\, \mathrm{e}^{-\xi}\xi^{1/2} L_n^{1/2}(\xi) L_m^{1/2}(\xi) = \begin{cases} 0 & (m \neq n) \\ \frac{\Gamma(n+3/2)}{\Gamma(n+1)} & (m = n). \end{cases} \tag{S32}$$

We first treat a simple case in which the promoter is located at $z = 0$ at $t = 0$, $\psi_0(z) = \delta(z)$. In this case, the coefficients $C_n(0)$ are given by

$$C_n(0) = \frac{2}{\sqrt{\pi}}, \tag{S33}$$

where we used the relationship

$$L_n^{1/2}(0) = \frac{2}{\sqrt{\pi}} \frac{\Gamma(n+3/2)}{\Gamma(n+1)}. \tag{S34}$$

Substituting eq. (S33) into eq. (S30) leads to the form

$$\begin{aligned} \psi(z,t)dz &= \frac{2}{\sqrt{\pi}} \sum_{n=0}^{\infty} L_n^{1/2}(\xi)\xi^{1/2}\mathrm{e}^{-\xi}\mathrm{e}^{-\omega_n t} d\xi \\ &= \frac{2}{\sqrt{\pi}} \frac{\xi^{1/2} d\xi}{(1-\mathrm{e}^{-t/\tau_{\mathrm{p}}})^{3/2}} \mathrm{e}^{-\xi/(1-\mathrm{e}^{-t/\tau_{\mathrm{p}}})} \\ &= \frac{4}{\sqrt{\pi}} \frac{z^2 dz}{(2l_{\mathrm{p}}^2 r_{\mathrm{s}}(t))^{3/2}} \mathrm{e}^{-z^2/(2l_{\mathrm{p}}^2 r_{\mathrm{s}}(t))}, \end{aligned} \tag{S35}$$

with

$$r_{\mathrm{s}}(t) = 1 - \mathrm{e}^{-t/\tau_{\mathrm{p}}}. \tag{S36}$$

The relaxation time $\tau_{\rm p}$ has the form

$$\tau_{\rm p} = \frac{N\zeta l_{\rm p}^2}{2k_{\rm B}T}. \tag{S37}$$

We used $\omega_n = n/\tau_{\rm p}$ and the generating function of the Laguerre polynomials

$$\sum_{n=0}^{\infty} L_n^{1/2}(\xi)x^n = \frac{1}{(1-x)^{3/2}}\mathrm{e}^{-\xi x/(1-x)} \tag{S38}$$

to derive the second line of eq. (S35).

With loop extrusion present, the time evolution equation of the relaxation process is given by

$$\begin{aligned} &\frac{\partial}{\partial t}\psi(z,t;N,N_{\rm p}) + \frac{1}{\tau_{\rm m}}\frac{\partial}{\partial N_{\rm p}}\psi(z,t;N,N_{\rm p}) \\ = \ &\frac{k_{\rm B}T}{N\zeta}\frac{\partial}{\partial z}\left[\frac{\partial}{\partial z}\psi(z,t;N,N_{\rm p}) + \frac{\partial}{\partial z}\left(\frac{U_{\rm eff}(z,t)}{k_{\rm B}T}\right)\psi(z,t;N,N_{\rm p})\right], \end{aligned} \tag{S39}$$

see eq. (10) in the main article. By using the characteristic curves

$$\frac{dN(t)}{dt} = 0 \tag{S40}$$

$$\frac{dN_{\rm p}(t)}{dt} = \frac{1}{\tau_{\rm m}}, \tag{S41}$$

eq. (S39) is rewritten as

$$\frac{\partial}{\partial t}\psi(z,t;N,N_{\rm p}(t)) = \frac{k_{\rm B}T}{N\zeta}\frac{\partial}{\partial z}\left[\frac{\partial}{\partial z}\psi(z,t;N,N_{\rm p}(t)) + \frac{\partial}{\partial z}\left(\frac{U_{\rm eff}(z,t)}{k_{\rm B}T}\right)\psi(z,t;N,N_{\rm p}(t))\right]. \tag{S42}$$

The solutions of eqs. (S40) and (S41) are $N = N_0$ and eq. (S16).

We derive the solution of eq. (S42) in the form

$$\psi(z,t) \;=\; \frac{4}{\sqrt{\pi}}\frac{z^2}{(2l_{\rm eq}^2 r_{\rm p}(t))^{3/2}}{\rm e}^{-z^2/(2l_{\rm eq}^2 r_{\rm p}(t))}. \tag{S43}$$

Substituting eq. (S43) into eq. (S42) leads to the form

$$\text{(Left side)} \;=\; \left(\frac{d}{dt}r_{\rm p}(t)\right)\left[-\frac{3}{2}\frac{1}{r_{\rm p}}+\frac{1}{2}\frac{z^2}{l_{\rm eq}^2 r_{\rm p}^2}\right]\psi(z,t) \tag{S44}$$

$$\text{(Right side)} \;=\; -\frac{2}{l_{\rm eq}^2}\frac{k_{\rm B}T}{N\zeta}\left(\frac{l_{\rm eq}^2}{l_{\rm p}^2}r_{\rm p}-1\right)\left[-\frac{3}{2}\frac{1}{r_{\rm p}}+\frac{1}{2}\frac{z^2}{l_{\rm eq}^2 r_{\rm p}^2}\right]\psi(z,t). \tag{S45}$$

Eq. (S42) is thus reduced to

$$\frac{d}{dt}r_{\rm p}(t) = -\frac{2}{l_{\rm eq}^2}\frac{k_{\rm B}T}{N\zeta}\left(\frac{l_{\rm eq}^2}{l_{\rm p}^2}r_{\rm p}(t)-1\right). \tag{S46}$$

The solution of eq. (S46) has the form

$$r_{\rm p}(t) \;=\; \frac{2k_{\rm B}T}{N_0\zeta l_{\rm eq}^2}\int_0^t dt_2\,{\rm e}^{-\int_{t_2}^t dt_1\,\frac{2k_{\rm B}T}{N_0\zeta l_{\rm p}^2(t_1)}}, \tag{S47}$$

where we used $r_{\rm p}(0) = 0$ for the initial condition (corresponding to $\psi(z,0) = \delta(z)$).

S3 Probability distribution function in loop extrusion process

In the loop extrusion process, the time evolution equation of the probability distribution function has the form

$$\begin{aligned}&\frac{\partial}{\partial t}\psi(z,t;N,N_{\rm p}) - \frac{1}{\tau_{\rm m}}\frac{\partial}{\partial N}\psi(z,t;N,N_{\rm p}) + \frac{1}{\tau_{\rm m}}\frac{\partial}{\partial N_{\rm p}}\psi(z,t;N,N_{\rm p})\\ &= \frac{k_{\rm B}T}{N_0\zeta}\frac{\partial}{\partial z}\left[\frac{\partial}{\partial z}\psi(z,t;N,N_{\rm p}) + \frac{\partial}{\partial z}\left(\frac{U_{\rm eff}(z,t)}{k_{\rm B}T}\right)\psi(z,t;N,N_{\rm p})\right].\end{aligned} \tag{S48}$$

By using the characteristic curves

$$\frac{dN_{\mathrm{p}}(t)}{dt} = \frac{1}{\tau_{\mathrm{m}}} \tag{S49}$$

$$\frac{dN(t)}{dt} = -\frac{1}{\tau_{\mathrm{m}}}, \tag{S50}$$

eq. (S48) is rewritten in the form

$$\frac{\partial}{\partial t}\psi(z,t;N(t),N_{\mathrm{p}}(t)) = \frac{k_{\mathrm{B}}T}{N_0\zeta}\frac{\partial}{\partial z}\left[\frac{\partial}{\partial z}\psi(z,t;N(t),N_{\mathrm{p}}(t)) + \frac{\partial}{\partial z}\left(\frac{U_{\mathrm{eff}}(z,t)}{k_{\mathrm{B}}T}\right)\psi(z,t;N(t),N_{\mathrm{p}}(t))\right]. \tag{S51}$$

The solutions of eqs. (S49) and (S50) are eqs. (S16) and (S17), respectively.

We derive the solution of eq. (S51) in the form

$$\psi(z,t) = \frac{4}{\sqrt{\pi}}\frac{z^2}{(2l_{\mathrm{eq}}^2 r_{\mathrm{ex}}(t'))^{3/2}}\mathrm{e}^{-z^2/(2l_{\mathrm{eq}}^2 r_{\mathrm{ex}}(t'))}, \tag{S52}$$

where t' $(= t - t_0)$ is the time elapsed after the loop extrusion starts, see also eq. (S35). Substituting eq. (S52) into eq. (S51) leads to the form

$$(\text{Left side}) = \left(\frac{d}{dt}r_{\mathrm{ex}}\right)\left[-\frac{3}{2}\frac{1}{r_{\mathrm{ex}}} + \frac{1}{2}\frac{z^2}{l_{\mathrm{eq}}^2 r_{\mathrm{ex}}^2}\right]\psi(z,t) \tag{S53}$$

$$(\text{Right side}) = -\frac{2}{l_{\mathrm{eq}}^2}\frac{k_{\mathrm{B}}T}{N\zeta}\left(\frac{l_{\mathrm{eq}}^2}{l_{\mathrm{p}}^2}r_{\mathrm{ex}} - 1\right)\left[-\frac{3}{2}\frac{1}{r_{\mathrm{ex}}} + \frac{1}{2}\frac{z^2}{l_{\mathrm{eq}}^2 r_{\mathrm{ex}}^2}\right]\psi(z,t). \tag{S54}$$

Eq. (S52) is finally reduced to

$$\frac{d}{dt}r_{\mathrm{ex}}(t) = -\frac{2}{l_{\mathrm{eq}}^2}\frac{k_{\mathrm{B}}T}{N\zeta}\left(\frac{l_{\mathrm{eq}}^2}{l_{\mathrm{p}}^2(t)}r_{\mathrm{ex}}(t) - 1\right). \tag{S55}$$

The solution of eq. (S55) has the form

$$r_{\mathrm{ex}}(t') = r_{\mathrm{p}}(t_0)\mathrm{e}^{-\int_0^{t'} dt_1 \frac{2k_{\mathrm{B}}T}{N(t_1)\zeta l_{\mathrm{p}}^2(t_1)}} + \int_0^{t'} dt_2 \frac{2k_{\mathrm{B}}T}{N(t_2)\zeta l_{\mathrm{eq}}^2}\mathrm{e}^{\int_{t'}^{t_2} dt_1 \frac{2k_{\mathrm{B}}T}{N(t_1)\zeta l_{\mathrm{p}}^2(t_1)}}, \tag{S56}$$

where we set $r_{\mathrm{ex}}(t' = 0) = r_{\mathrm{p}}(t_0)$ for the initial condition.

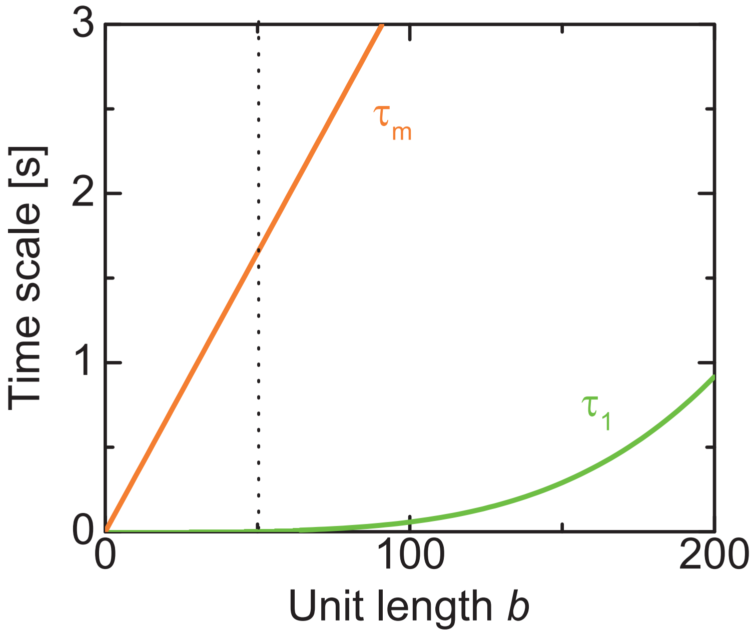

Figure S1: The time scale τ_1 of the relaxation of each chain unit and the time scale τ_{m} of the loop extrusion of one unit by cohesin are shown as functions of the unit length b. This estimate corresponds to the case in which nucleosomes are linked by bare DNA of length 19 bps.[1] DNA of length 146 bps is wound around an histone octamer in each nucleosome. We estimated τ_1 by using the mean square displacement of nucleosomes in HeLa cells ($8b^2/(3\pi^{3/2}\sqrt{\tau_1}) \approx 0.02\ \mu\mathrm{m}^2/\mathrm{s}^{1/2}$).[2]

References

(1) Socol,M., Wang,R., Jost,D., Carrivain, P., Vaillant,C., Cam,E.L., Dahirel,V., Normand,C., Bystricky,K., Victor,J.M., Gadal,O., and Bancaud,A. () Rouse model with transient intramolecular contacts on a timescale of seconds recapitulates folding and fluctuation of yeast chromosomes. *Nucleic Acid Res.*, **47,** 6195–6207.

(2) Nozaki,T., Imai,R., Tanbo,M., Nagashima,R., Tamura,S., Tani,T., Joti,Y., Tomita,M., Hibino,K., Kanemaki,M.T., Wendt,K.S., Okada,Y., Nagai,T., Maeshima,K. (2017) Dynamic Organization of Chromatin Domains Revealed by Super-Resolution Live-Cell Imaging. *Mol Cell*, **67**, 282-293.
